# The PD-1/PD-L1 pathway is induced during *Borrelia burgdorferi* infection and inhibits T cell joint infiltration without compromising bacterial clearance

**DOI:** 10.1371/journal.ppat.1010903

**Published:** 2022-10-20

**Authors:** Jennifer D. Helble, Julie E. McCarthy, Machlan Sawden, Michael N. Starnbach, Linden T. Hu

**Affiliations:** 1 Department of Molecular Biology and Microbiology, Tufts University School of Medicine, Boston, Massachusetts, United States of America; 2 Department of Microbiology, Harvard Medical School, Boston, Massachusetts, United States of America; University of Montana, UNITED STATES

## Abstract

The Lyme disease bacterial pathogen, *Borrelia burgdorferi*, establishes a long-term infection inside its mammalian hosts. Despite the continued presence of the bacteria in animal models of disease, inflammation is transitory and resolves spontaneously. T cells with limited effector functions and the inability to become activated by antigen, termed exhausted T cells, are present in many long-term infections. These exhausted T cells mediate a balance between pathogen clearance and preventing tissue damage resulting from excess inflammation. Exhausted T cells express a variety of immunoinhibitory molecules, including the molecule PD-1. Following *B*. *burgdorferi* infection, we found that PD-1 and its ligand PD-L1 are significantly upregulated on CD4^+^ T cells and antigen presenting cell subsets, respectively. Using mice deficient in PD-1, we found that the PD-1/PD-L1 pathway did not impact bacterial clearance but did impact T cell expansion and accumulation in the ankle joint and popliteal lymph nodes without affecting B cell populations or antibody production, suggesting that the PD-1/PD-L1 pathway may play a role in shaping the T cell populations present in affected tissues.

## Author summary

Lyme disease is caused by the bacterial pathogen *Borrelia burgdorferi* which can establish a long-term infection inside its mammalian hosts. One hallmark of other long-term infections is the presence of T cells that express immunoinhibitory molecules, such as PD-1. Here, we sought to characterize if the PD-1/PD-L1 pathway was upregulated during murine *B*. *burgdorferi* infection and to understand its role in the outcome of infection. We demonstrated that the PD-1/PD-L1 pathway is enriched following *B*. *burgdorferi* infection and while PD-1^-/-^ mice do not exhibit a reduction in bacterial load compared to wild-type controls, they do exhibit an increased influx of T cells to the tissues following infection without affecting the B cell response. Our study suggests that the PD-1/PD-L1 pathway represents a key checkpoint to T cell expansion and tissue infiltration during Lyme disease.

## Introduction

The bacterial pathogen *Borrelia burgdorferi*, the causative agent of Lyme disease, is able to establish a long-term infection inside its mammalian hosts [1]. If left untreated, infection in humans can lead to severe inflammatory consequences including carditis, meningitis, and arthritis. As *B*. *burgdorferi* does not produce molecules that directly induce tissue destruction, host damage is thought to be mediated by the immune response [2]. As such, the immune system plays a vital role in mediating the balance between bacterial clearance and excessive inflammation.

Interestingly, patients that develop more severe manifestations of Lyme disease are usually able to resolve this inflammation even in the absence of antibiotic treatment [3]. This is also true in animal models of disease, where despite the continued presence of the bacteria, inflammation is transitory and resolves spontaneously [4]. While *B*. *burgdorferi* is able to evade antibody mediated killing by changing expression of key targets such as VlsE [2,5,6], it is unclear why this evasion also results in resolution of inflammatory responses. One possible mechanism is through built in checkpoints in the immune system that can dampen inflammatory responses if they are more damaging than the pathogen they were originally targeting.

T cells are a critical component of the adaptive immune response and have been shown to play an important role in both ameliorating infection and causing the pathology associated with *B*. *burgdorferi* infection. Early work in mice demonstrated the critical role of CD4^+^ T cells in resolving murine Lyme-associated joint arthritis [7] and carditis [8], and that T-independent B cell responses are important for resolving inflammation and generating protective antibodies [9]. However, both CD4^+^ and CD8^+^ T cells have also been shown to increase the severity of murine Lyme arthritis [10,11]. Despite the importance of CD4^+^ T cells in controlling spirochete levels in mouse skin, joint, bladder and heart [7,12], *Borrelia* is maintained in mice for at least one year following initial infection [4]. One possible explanation is that the immune system shifts from a pro-inflammatory state to an anti-inflammatory state by promoting the formation of an exhausted CD4^+^ T cell population. This would result in decreased inflammatory tissue damage, but at the cost of incomplete eradication of the organism.

Exhausted T cells are common in many long-term bacterial [13–16] and viral infections [17–23] and are characterized by their upregulation of immunoinhibitory molecules, such as the receptor protein PD-1. These T cells are specifically impaired in their ability to be activated by antigen, resulting in reduced T cell effector function and inadequate pathogen clearance [24]. Immunoinhibitory molecules present on CD4^+^ and CD8^+^ T cells interact with ligands such as PD-L1 on professional antigen presenting cells or other non-hematopoietic cells [25]. While PD-1 is commonly upregulated through repeated T cell receptor signaling, PD-L1 expression has been shown to be inducible through both type I (IFN-α and IFN-β) and type II interferons (IFN-γ) [25–27], both of which play an important role in the development of murine Lyme arthritis and human disease, respectively [28–30].

In this study, we sought to determine if *B*. *burgdorferi* infection could induce upregulation of the PD-1/PD-L1 immunoinhibitory pathway and to identify its subsequent role on the outcome of infection. We found that PD-L1 was upregulated on macrophages and CD11b^+^ CD103^-^ dendritic cells following infection, correlating with the observed upregulation of PD-1 on CD4^+^ T cells. In PD-1^-/-^ mice that are deficient in the PD-1/PD-L1 pathway, we observed no differences in bacterial load, inflammatory cytokines or B cell responses, but did observe increases in T cell infiltration to the draining lymph nodes and ankle joints. These data suggest that while the PD-1/PD-L1 pathway does not play a role in promoting *B*. *burgdorferi* longevity in a mammalian host, it does act to limit T cell accumulation.

## Results

### *Borrelia burgdorferi* stimulation induces PD-L1 upregulation on macrophages and dendritic cells

**Fig 1:**
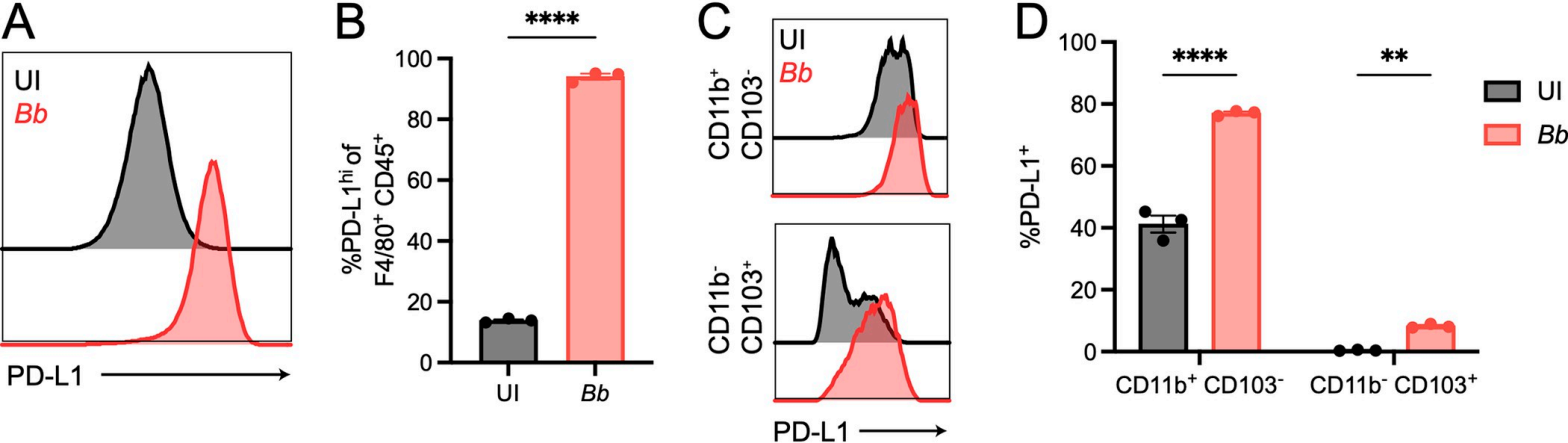
The immunoinhibitory ligand PD-L1 is upregulated *in vitro* following *Borrelia burgdorferi* stimulation. (A-B) Bone marrow derived macrophages (BMDMs) and (C-D) bone marrow derived dendritic cells (BMDCs) from wild-type (WT; C57BL/6) mice were stimulated with *B*. *burgdorferi* for 24 hours at an MOI of 10 and assessed for PD-L1 expression by flow cytometry. Data are representative of at least two independent experiments with three technical replicates and were analyzed using unpaired *t* test (B) or two-way analysis of variance (ANOVA) with Sidak’s multiple comparisons test (D).

To determine if *B*. *burgdorferi* stimulation promotes PD-L1 upregulation on antigen presenting cells, we generated bone marrow derived macrophages (BMDMs) and dendritic cells (BMDCs) from wild-type (WT) C57BL/6 mice. These cells were stimulated for 24 hours with *B*. *burgdorferi* before determining PD-L1 expression by flow cytometry (S1A and S1B Fig). We found that *B*. *burgdorferi*-stimulated BMDMs significantly upregulated PD-L1 compared to uninfected controls (Fig 1A and 1B), suggesting that macrophage stimulation by *B*. *burgdorferi* infection can induce upregulation of immunoinhibitory molecules. While there are many subsets of dendritic cells [31], we chose to focus on two conventional dendritic cell subsets, CD11b^+^ CD103^-^ cells and CD11b^-^ CD103^+^ cells (S1B Fig) [32]. We found that both *B*. *burgdorferi*-stimulated CD11b^+^ CD103^-^ BMDCs and CD11b^-^ CD103^+^ BMDCs significantly upregulated PD-L1 compared to uninfected controls, although the levels of PD-L1 upregulation on CD11b^-^ CD103^+^ BMDCs were substantially lower than on CD11b^+^ CD103^-^ BMDCs (Fig 1C and 1D). Taken together, these data suggest that *B*. *burgdorferi* stimulation induces PD-L1 upregulation on bone marrow derived antigen presenting cells.

**Fig 2:**
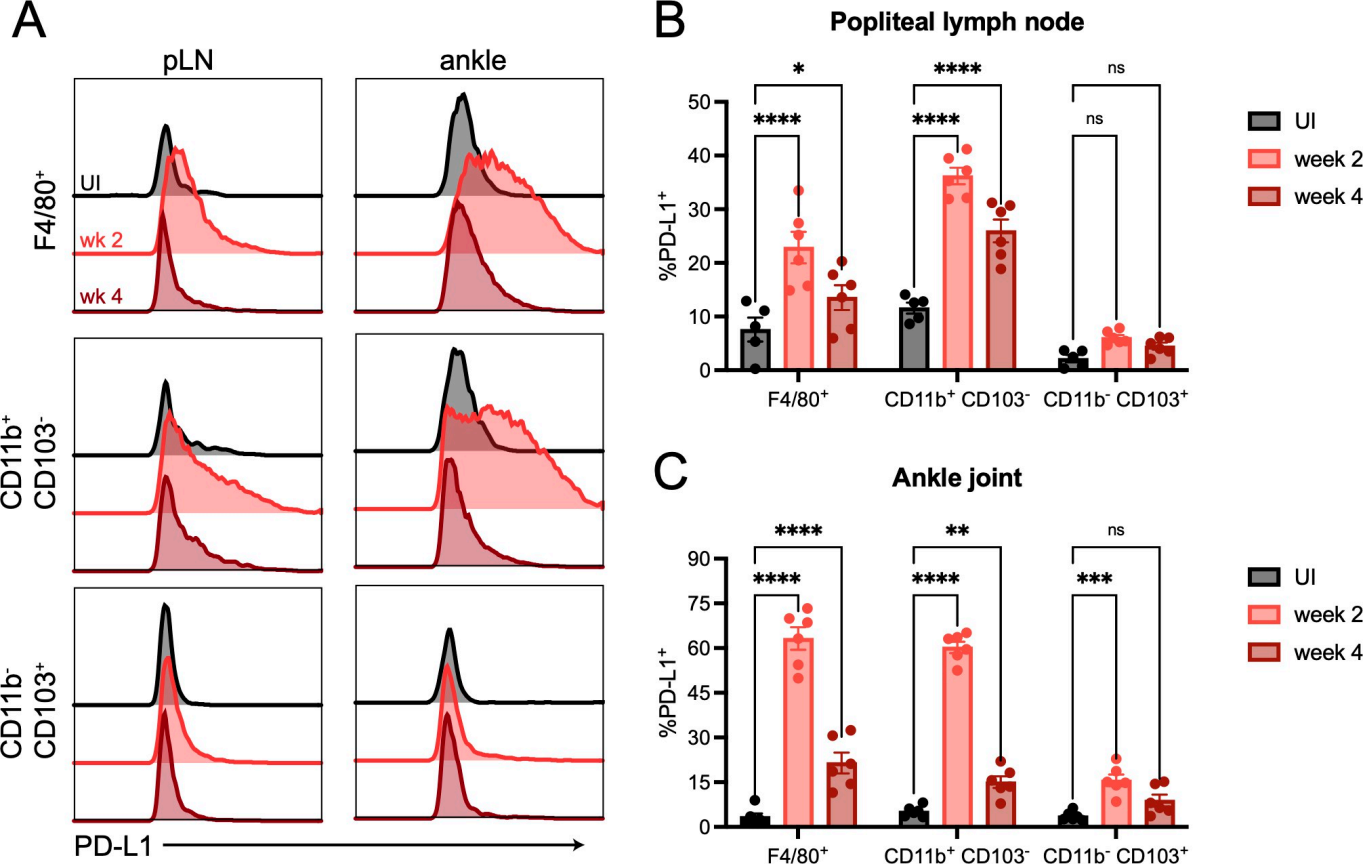
PD-L1 is upregulated on macrophages and CD11b^+^ CD103^-^ dendritic cells in the draining lymph nodes and ankle joints following murine *B*. *burgdorferi* infection. WT mice were inoculated with *B*. *burgdorferi* or media alone and sacrificed at two and four weeks post-infection. (A, B) Popliteal lymph nodes and (A, C) ankle joints were harvested and assessed for flow cytometry. Data are pooled from two independent experiments with at least 5 mice per group and were analyzed using two-way ANOVA with Dunnett’s multiple comparisons test.

While BMDMs and BMDCs can be a good proxy for macrophages and dendritic cells *in vivo*, respectively, it is unclear if *B*. *burgdorferi* infection in mice could induce a similar PD-L1 response. To test this, we infected WT mice with *B*. *burgdorferi* and monitored PD-L1 upregulation in F4/80^+^ macrophages, CD11b^+^ CD103^-^ dendritic cells and CD11b^-^ CD103^+^ dendritic cells at two and four weeks post-infection (S2A Fig). We specifically chose to look at cells infiltrating the ankle joints, a common site of inflammation following *B*. *burgdorferi* infection, and the popliteal lymph nodes that drain the ankle joint. In both the popliteal lymph nodes (Fig 2A and 2B) and ankle joint (Fig 2A and 2C), we found that PD-L1 expression on F4/80^+^ macrophages and CD11b^+^ CD103^-^ dendritic cells peaked in infected mice at two weeks post-infection and was still significantly elevated above uninfected controls at four weeks post-infection. CD11b^-^ CD103^+^ dendritic cells in the popliteal lymph node did not upregulate PD-L1 compared to uninfected controls, although PD-L1 expression on CD11b^-^ CD103^+^ dendritic cells in the ankle joint were elevated above baseline only at week two post-infection. We also found that in the inguinal lymph nodes which drain the injection site, PD-L1 was upregulated on CD11b^+^ CD103^-^ dendritic cells following infection (S2B Fig). Overall, our *in vivo* results validate our *in vitro* observations, and suggest that there are specific antigen presenting cell subsets that upregulate PD-L1 following *B*. *burgdorferi* infection.

### *B*. *burgdorferi* infection induces PD-1 upregulation on CD4^+^ T cells

**Fig 3:**
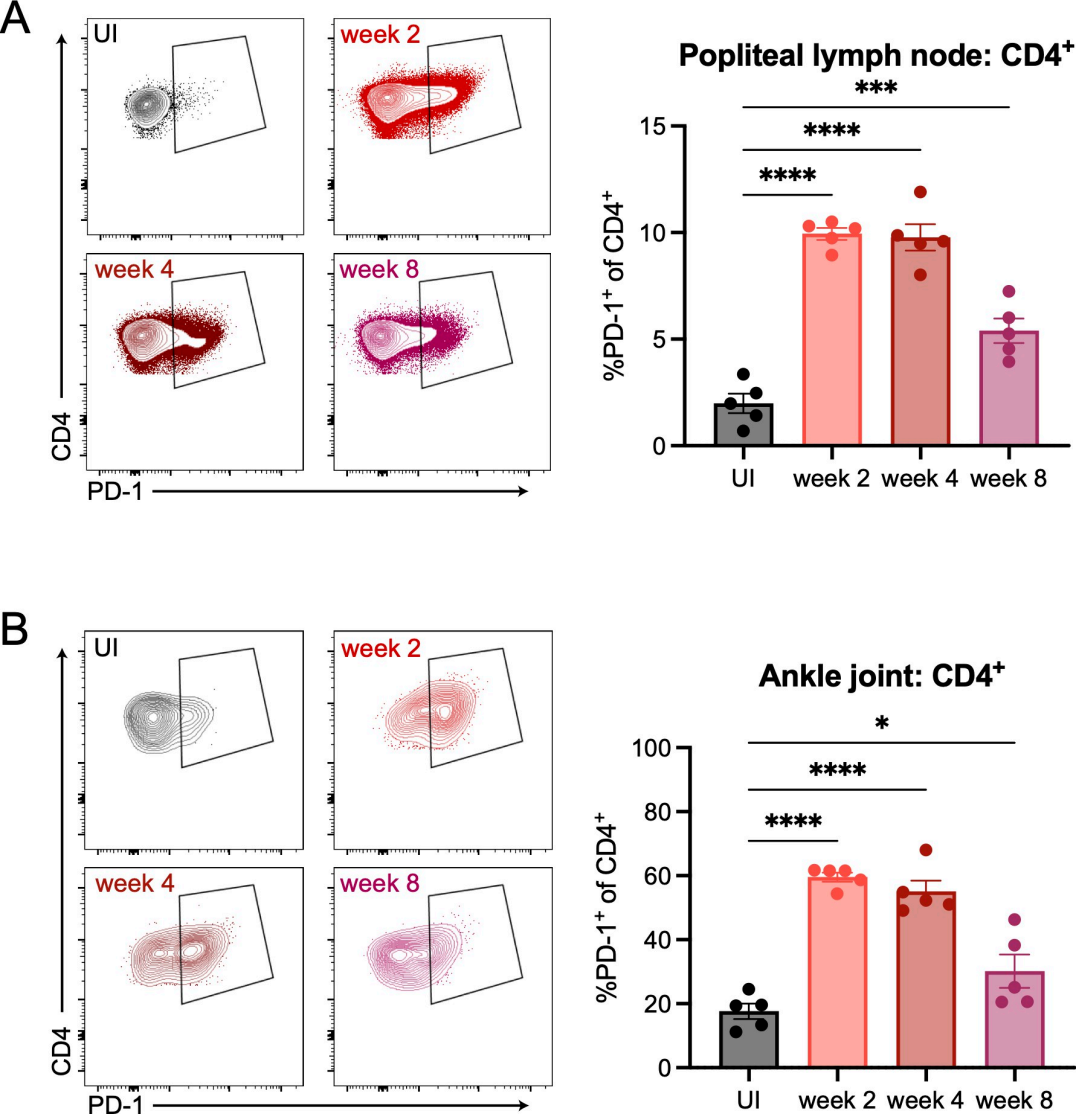
PD-1 is specifically upregulated on CD4^+^ T cells in the draining lymph nodes and ankle joints following *B*. *burgdorferi* infection. WT mice were inoculated with *B*. *burgdorferi* or media alone and sacrificed at two, four, and eight weeks post-infection. Popliteal lymph nodes (A) and ankle joints (B) were isolated and assessed by flow cytometry for PD-1 expression on CD4^+^ T cells. Data are representative of four independent experiments, shown with five mice per group. Data were analyzed using ordinary one-way ANOVA with Dunnett’s multiple comparisons test.

PD-L1 on antigen presenting cells can interact with PD-1 on T cells. We next wanted to test if PD-1 was upregulated on T cells following *B*. *burgdorferi* infection. Therefore, we infected WT mice and assessed PD-1 expression by flow cytometry following infection (S3A Fig). In the popliteal lymph node of mice, we found that CD4^+^ T cells (Fig 3A) but not CD8^+^ T cells or B cells (S3B Fig) specifically upregulated PD-1 at two, four, and eight weeks post-infection compared to uninfected controls. Similarly, we observed PD-1 upregulation on CD4^+^ T cells (Fig 3B) but not CD8^+^ T cells or B cells (S3C Fig) in the ankle joints two, four, and eight weeks post-infection. Inguinal lymph node CD4^+^ T cells but not CD8^+^ T cells or B cells also upregulated PD-1 following infection (S3D Fig). These data suggest that PD-1 upregulation during *B*. *burgdorferi* infection does not occur across all T cell subsets, but is specifically localized to the CD4^+^ T cell compartment.

### PD-1 expression is primarily found on T-bet^+^ CD4^+^ T cells and effector memory CD4^+^ T cells

**Fig 4:**
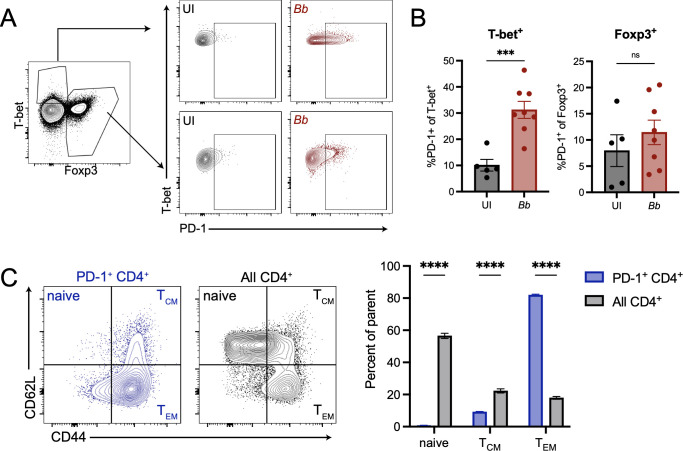
PD-1 upregulation is primarily found on T-bet^+^ CD4^+^ T cells and not Foxp3^+^ CD4^+^ T cells. WT mice were inoculated with *B*. *burgdorferi* or media alone and sacrificed at four weeks post-infection. Popliteal lymph nodes were harvested and CD4^+^ T cells were assessed by flow cytometry for expression of (A, B) Foxp3 and T-bet through gating on live ➔ CD3^+^ ➔ CD4^+^ ➔ Foxp3^+^ or T-bet^+^ cells followed by PD-1^+^ gating. (C) Popliteal lymph nodes were separately harvested and PD-1^+^ CD4^+^ T cells (blue) and total CD4^+^ T cells (gray) were assessed for effector memory (T_EM_), central memory (T_CM_) or naïve status based on CD44/CD62L staining. Data are representative of two independent experiments with at least 5 mice per group and were analyzed using (B) unpaired *t* test or (C) two-way ANOVA with Sidak’s multiple comparisons test.

We next wanted to determine the main PD-1 expressing CD4^+^ T cell subset during *B*. *burgdorferi* infection. Foxp3^+^ regulatory T cells (Tregs) have been shown in other systems to express high levels of PD-1 [33–36], and we wanted to determine if the increased PD-1 expression in infected mice was due to changes in the Treg population or to other T cell subsets. We specifically chose to compare Treg PD-1 expression to that of T-bet^+^ Th1 CD4^+^ T cells, which have been strongly implicated in Lyme disease pathogenesis [30,37,38]. When we examined PD-1 upregulation on Foxp3^+^ or T-bet^+^ T cells in the popliteal lymph node (Fig 4A), we found that Foxp3^+^ CD4^+^ T cells did not increase PD-1 expression in four week infected mice compared to uninfected controls (Fig 4B, right). However, T-bet^+^ CD4^+^ T cells in four week infected mice significantly upregulated PD-1 compared to uninfected controls (Fig 4B, left), suggesting that the PD-1 upregulation we had observed (Fig 3) was not driven by Foxp3^+^ Tregs, but rather T-bet^+^ Th1 cells. We also found that PD-1^+^ CD4^+^ T cells were mainly comprised of CD44^+^ CD62L^-^ effector memory cells compared to total CD4^+^ T cells, which were mainly naïve (CD44^-^ CD62L^+^) (Fig 4C).

### Importance of PD-1 in controlling *B*. *burgdorferi* loads and Lyme arthritis

**Fig 5:**
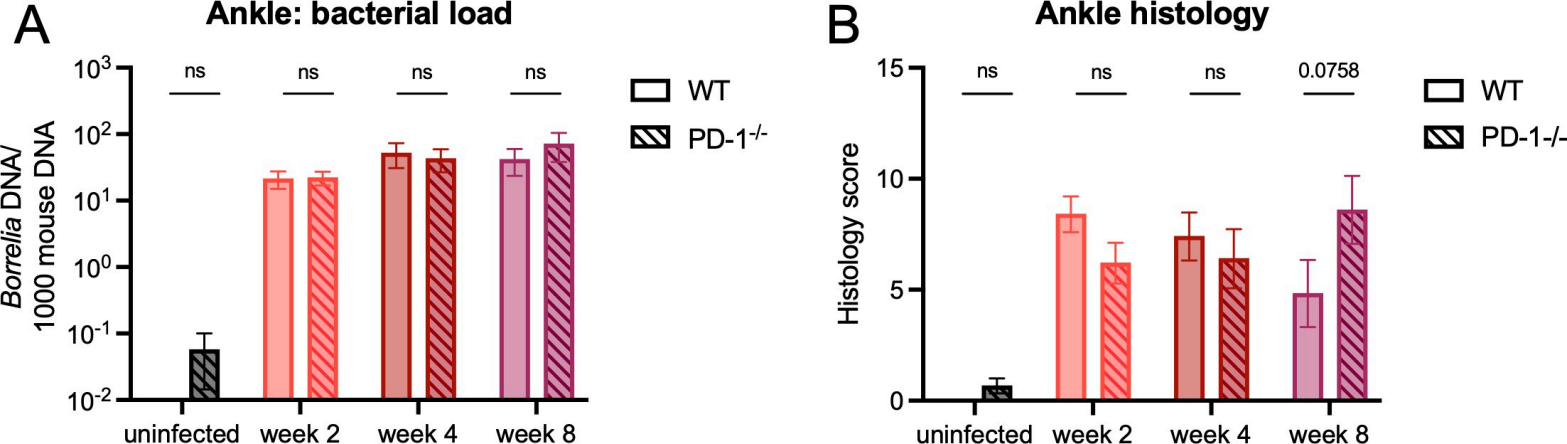
The role of PD-1 in controlling *B*. *burgdorferi* loads and Lyme arthritis. WT and PD-1^-/-^ mice were inoculated with *B*. *burgdorferi* or media alone and sacrificed at two, four, and eight weeks post-infection. (A) DNA was isolated from ankle joints and bacterial load was assessed by ddPCR. (B) Ankle joints were fixed, sectioned and stained with H&E and scored blindly by a pathologist for synovitis, tenosynovitis/tendonitis, periarticular fibrosis, synovial proliferation, articular cartilage degeneration or erosion, periosteal remodeling, and effusion/edema. Data are representative of three experiments pooled with at least five mice per infected group and were analyzed using (A) two-way ANOVA with Sidak’s multiple comparisons test and (B) Mann-Whitney test.

T cells expressing immunoinhibitory molecules such as PD-1, can prevent adequate pathogen clearance due to their inability to become activated and produce proinflammatory cytokines [27,39]. Given that *B*. *burgdorferi* can survive in hosts for long periods of time, we hypothesized that the PD-1^+^ CD4^+^ T cell population would promote *B*. *burgdorferi* longevity and that removal of the PD-1/PD-L1 pathway would promote enhanced bacterial clearance. To test this, we infected WT and PD-1^-/-^ mice with *B*. *burgdorferi* and assessed bacterial burden using droplet digital PCR (ddPCR) at two, four, and eight weeks post-infection. We found that bacterial load was unchanged in WT and PD-1^-/-^ mice in the ankles (Fig 5A) and ears (S4A Fig). Bacterial load was also largely unchanged in the heart (S4B Fig) at later time points and slightly higher in PD-1^-/-^ mice at two weeks post-infection. These data suggest that irrespective of anatomical location, the PD-1/PD-L1 pathway does not play a critical role in controlling murine *B*. *burgdorferi* infection *in vivo*.

While the PD-1/PD-L1 pathway can be important for bacterial control, it has also been shown to regulate inflammation in the context of other infections. We assessed histology in the ankle joints of WT and PD-1^-/-^ mice at all three time points and found that while there were no significant differences in overall score, PD-1^-/-^ mice at week eight post-infection did have slightly higher scores than WT mice (Fig 5B). Furthermore, ankle histology scores for WT mice tended to decrease over time, while scores from PD-1^-/-^ mice slightly increased, suggesting that the PD-1/PD-L1 pathway may be responsible for limiting ankle inflammation during *B*. *burgdorferi* infection.

Removal or inhibition of the PD-1/PD-L1 pathway can also induce enhanced proinflammatory cytokine production [14,19,25]. Even though WT and PD-1^-/-^ mice were able to control *B*. *burgdorferi* infection equally well, we hypothesized that there may have been differences in cytokine production that ultimately did not manifest as differences in bacterial load. To test this, we assessed the production of 31 different proinflammatory cytokines in the ankle joints of WT and PD-1^-/-^ mice (S5A Fig). Out of the 31 cytokines assayed, only CCL4 (S5B Fig) and CXCL1 (S5C Fig) were heightened in infected PD-1^-/-^ mice compared to WT mice. CCL4 and CXCL1 signal to induce influx of macrophages and neutrophils, respectively, and we therefore assessed if there was differential recruitment in macrophages and neutrophils in PD-1^-/-^ mice following *B*. *burgdorferi* infection. We found that while there were significantly more macrophages (S6A) and neutrophils (S6B) in the popliteal lymph nodes of PD-1^-/-^ mice (S6A Fig), this was limited to two weeks post-infection and did not overlap with the increased CCL4 and CXCL1 production we observed. There were also no significant differences in macrophage or neutrophil accumulation in the inguinal lymph node (S6C and S6D Fig) or macrophage accumulation in ankle joint (S6E). While there were significant differences in neutrophil accumulation in the ankle joint at week eight post-infection (S6F Fig), our data overall suggest that the increased cytokine production we observed did not necessarily impact cellular accumulation.

### PD-1 prevents T cell accumulation following *B*. *burgdorferi* infection without affecting B cell responses

**Fig 6:**
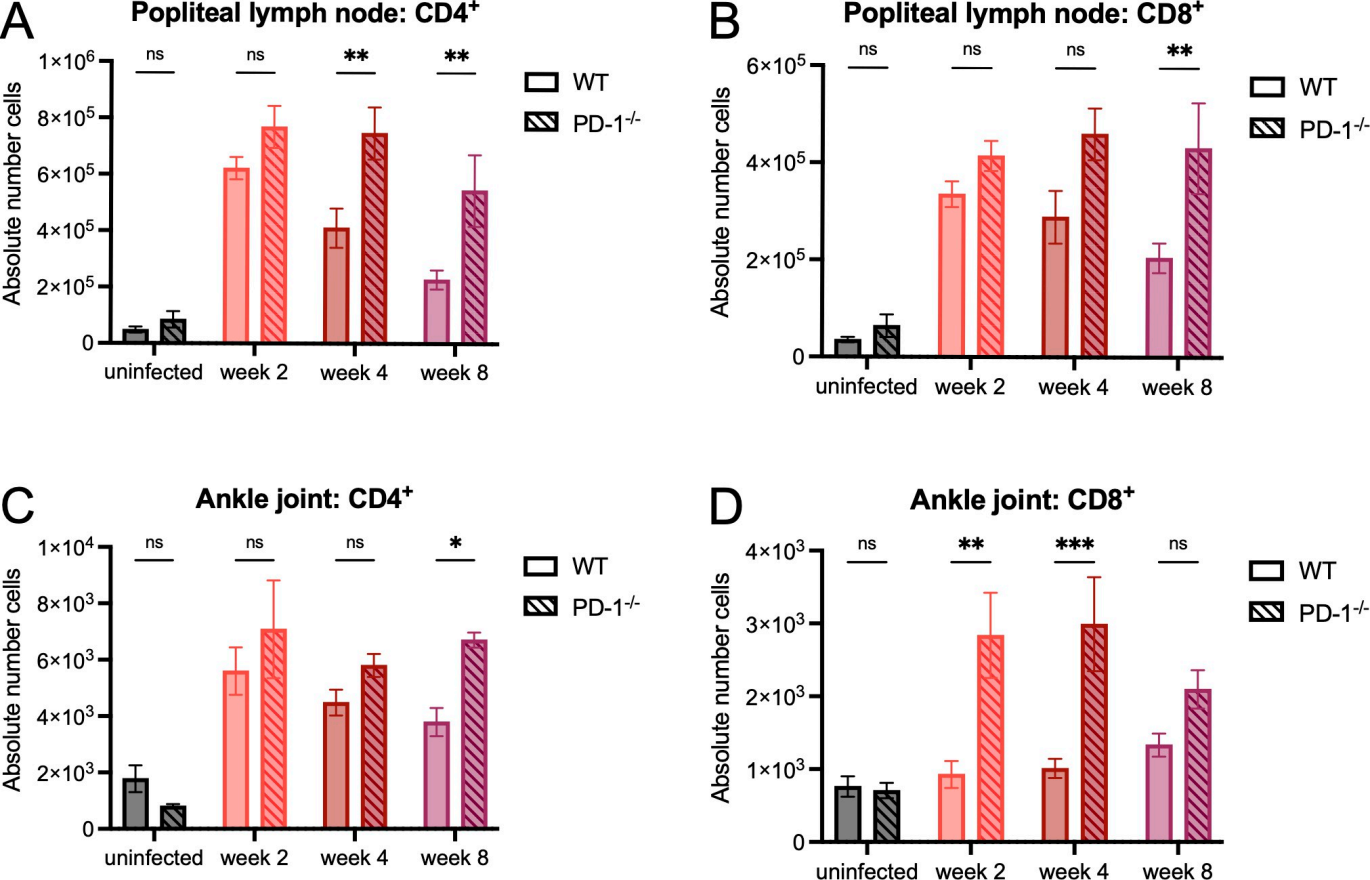
PD-1 prevents T cell accumulation during *B*. *burgdorferi* infection. WT and PD-1^-/-^ mice were inoculated with *B*. *burgdorferi* or media alone and sacrificed two, four, and eight weeks post-infection. Popliteal lymph nodes were assessed for accumulation of (A) CD4^+^ and (B) CD8^+^ T cells and ankle joints were assessed for accumulation of (C) CD4^+^ and (D) CD8^+^ T cells by flow cytometry through gating on live ➔ CD45^+^ CD3^+^ ➔ CD4^+^ or CD8^+^ cells. Data are pooled from four independent experiments with at least five mice per group and were analyzed using two-way ANOVA with Sidak’s multiple comparisons test.

Given the importance of PD-1 regulation on limiting T cell activation and expansion, we wanted to investigate if genetic ablation of PD-1 would result in any differences in T cell homing during *B*. *burgdorferi* infection. At two and four weeks post-infection, we assessed CD4^+^ and CD8^+^ T cell accumulation in the popliteal lymph nodes and ankle joints of WT and PD-1^-/-^ mice. Popliteal lymph nodes of PD-1^-/-^ mice at four and eight weeks post-infection contained significantly more CD4^+^ (Fig 6A) and at week eight contained significantly more CD8^+^ (Fig 6B) T cells than WT mice, suggesting that expression of PD-1 during *B*. *burgdorferi* infection prevents T cell accumulation in the lymph node. We also found in the ankle joints of PD-1^-/-^ mice, there was an increase in both CD4^+^ T cells at week eight post-infection (Fig 6C) and CD8^+^ T cells at weeks two and four post-infection (Fig 6D). We did not observe differences in CD4^+^ and CD8^+^ T cell accumulation in the inguinal lymph nodes (S7A and S7B Fig), suggesting that removal of the PD-1/PD-L1 pathway during *B*. *burgdorferi* infection does not impact all tissues.

**Fig 7:**
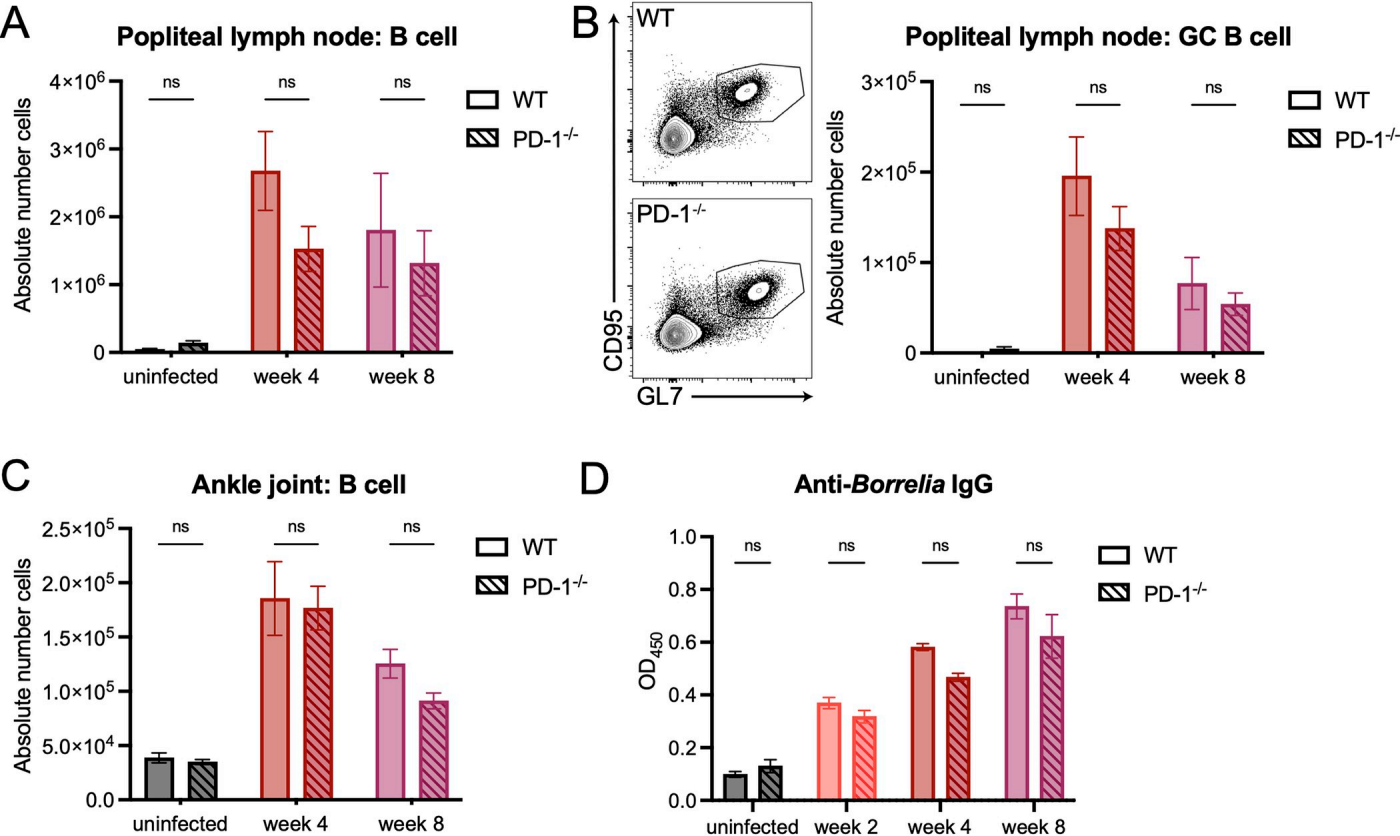
B cell responses to *B*. *burgdorferi* remain unchanged in the absence of PD-1. WT and PD-1^-/-^ mice were inoculated with *B*. *burgdorferi* or media alone. Popliteal lymph nodes were isolated and assessed by flow cytometry for total numbers of (A) B cells (live ➔ CD45^+^ B220^+^ cells) and (B) germinal center B cells (GL7^+^ CD95^+^ B cells). (C) Ankle joints were assessed for total B cells and (D) serum was assessed for anti-*Borrelia* IgG antibodies through ELISA. Data are pooled from at least two independent experiments with five mice per group and were analyzed using two-way ANOVA with Sidak’s multiple comparisons test.

During *B*. *burgdorferi* infection, B cells and the antibody response have been shown to be critical in responding to and eliminating the pathogen [9,40,41]. Given that increased levels of T cells in the ankle joint did not result in changes in bacterial load, we hypothesized that this was driven by an unchanged B cell response to *B*. *burgdorferi* in PD-1^-/-^ mice. To test this, we assessed total B cell populations in the popliteal (Fig 7A) and inguinal lymph nodes (S7C Fig) at four and eight weeks post-infection, and found that there were no significant differences in total B cell populations between WT and PD-1^-/-^ mice. We also found that removal of the PD-1/PD-L1 pathway did not impact germinal center B cell populations in both the popliteal (Fig 7B) and inguinal lymph nodes (S7D Fig). Ankle joint B cell populations also remained unchanged (Fig 7C). We also assessed serum antibody titers against *B*. *burgdorferi*, and found that at all time points, PD-1^-/-^ mice exhibited similar levels of anti-*Borrelia* IgG as WT mice (Fig 7D). Taken together, these data suggest that the PD-1/PD-L1 axis is critical for preventing T cell accumulation and represents an important checkpoint in the immune response to *B*. *burgdorferi* infection *in vivo*.

## Discussion

Persistent antigen stimulation induces T cell exhaustion in long-term infections and cancer. PD-1 along with its ligand, PD-L1, is a well characterized immunoinhibitory pathway that acts to limit T cell responses and has been shown to be upregulated during several long-term pathogens, including *Chlamydia trachomatis* [15], *Mycobacterium tuberculosis* [14], HIV [18], and LCMV [19]. Here, we sought to determine in mouse models if infection with the long-term bacterial pathogen *B*. *burgdorferi* upregulates the PD-1/PD-L1 pathway and to understand the role of this pathway in controlling bacterial load and cellular infiltration in the ankle joints and draining lymph nodes of mice.

We found that *in vitro* stimulation and *in vivo* infection with *B*. *burgdorferi* resulted in upregulation of PD-L1 on F4/80^+^ macrophages and CD11b^+^ CD103^-^ dendritic cells. Macrophages and dendritic cells are key professional antigen presenting cells that are important for presenting *Borrelia* protein antigen to T cells [40]. The two main dendritic cell subsets we chose to investigate, CD11b^-^ CD103^+^ (cDC1s) and CD11b^+^ CD103^-^ (cDC2s), differ significantly in their main functions. While cDC1s are important for immunity against intracellular pathogens and activating CD8^+^ T cell responses through presenting antigen on MHC I, cDC2s are critical for defense against extracellular pathogens and inducing CD4^+^ T cell immunity by presenting antigen on MHC II [32,42]. As *B*. *burgdorferi* is an extracellular pathogen, the enhanced PD-L1 upregulation on cDC2s aligns with the observed PD-1 expression on CD4^+^ T cells and likely indicates that cDC2s, along with macrophages, are possible antigen presenting cell populations that are critical for shaping the CD4^+^ T cell response to *B*. *burgdorferi*.

In other systems, PD-L1 has been shown to be inducible through the presence of either type I or type II interferon [27]. While we observed *B*. *burgdorferi* induced PD-L1 upregulation in both *in vitro* derived BMDMs and BMDCs as well as macrophages and cDC2s *in vivo*, it is unclear whether this was the result of IFN production. Given that IFN-γ is important for inducing Lyme arthritis in C57BL/6 mice [29], it is tempting to speculate that IFN-γ and not type I IFNs, which are more critical in C3H murine arthritis [28,43], was the main driver for PD-L1 upregulation *in vivo*.

While the contributions of T cells during *B*. *burgdorferi* infection have been heavily explored in the context of generating a protective B cell and antibody response, it is unclear whether the B cell-independent T cell responses are protective, pathologic, or impaired [40]. Our findings that *B*. *burgdorferi* infection leads to increased PD-1 expression on CD4^+^ T cells suggests there is some degree of impairment to the T cell compartment during infection. The observation that only the CD4^+^ T cell response is marked by increased PD-1 expression is perhaps not surprising, as *B*. *burgdorferi* is an extracellular pathogen and therefore the CD8^+^ T cell response is less critical. There are several different CD4^+^ T cell subsets, including various helper T cell subtypes and Foxp3^+^ regulatory T cells. Foxp3^+^ Tregs are important for maintaining tissue homeostasis often by secreting anti-inflammatory cytokines, and there have been several studies to suggest that Tregs during *B*. *burgdorferi* infection can be protective against excessive inflammation: in patients with antibiotic-refractory Lyme arthritis, higher numbers of Tregs in the joint synovial fluid correlated with faster resolution of arthritis symptoms [44,45]. Similarly, when mice depleted of Tregs were infected with *B*. *burgdorferi*, they experienced more severe arthritis compared to mice with a functional Tregs compartment [46]. Although Tregs can express high levels of PD-1 [33–36], we found that PD-1 expression driven by *B*. *burgdorferi* was not due to an increase in PD-1 expressing Foxp3^+^ cells but rather to T-bet^+^ Th1 cells. While the role of Th1 cells during Lyme disease and *Borrelia* pathogenesis is not completely clear, there are studies to suggest that Th1 cells are found in synovial fluid of patients with Lyme arthritis and that increased numbers of effector T cells correlates with a longer time to resolve arthritis [30,45,47]. Th17 cells have been implicated in human *B*. *burgdorferi* infection [48], but their responsiveness to murine infection varies depending on the model system [46,49,50] and we have not ruled out that PD-1 expression is also upregulated on Th17 cells in the C57BL/6 mouse model. PD-1 can inhibit T cell driven pro-inflammatory cytokine production in response to pathogens [14,19], however we only observed modest changes in cytokine production in the ankle joints following infection. It is possible that there are more localized changes in the cytokine milieu within the ankle joint, potentially corresponding to where the infection and subsequent T cell response occurs.

In other systems, removal of the PD-1/PD-L1 pathway promotes a reduction in pathogen load as a result of an expanded functional T cell population [15,18,19]. Interestingly, we found that in PD-1^-/-^ mice, there were no significant differences in *Borrelia* bacterial burden when compared to WT mice, suggesting that PD-1 expression does not control *B*. *burgdorferi* infection in mice. Given the importance of B cells and the antibody response in responding to and eliminating *B*. *burgdorferi* in mouse models of infection [9,40,41], removal of a primarily T cell pathway fits with a model of how B cell-driven immunity would not be significantly affected and would not result in changes in bacterial load. Indeed, germinal center T cells have been shown to highly express PD-1 upon *B*. *burgdorferi* infection, but this expression does not influence the ability of these T cells to form or maintain a germinal center response [12]. We did indeed find that PD-1^-/-^ mice exhibited similar numbers of B cells in both the popliteal lymph nodes and ankle joints following infection, and that germinal center B cell numbers remained unchanged. We also found that there was no difference in anti-*Borrelia* IgG production, suggesting that the PD-1/PD-L1 pathway does no influence antibody production. However, we have not ruled out production of other antibody isotypes or whether memory B cell formation is changed during *B*. *burgdorferi* infection in PD-1^-/-^ mice, as reports in the literature vary based on the model system [51]. Overall, however, our data suggest that PD-1 does not influence the B cell response, which in turn likely does not impact bacterial clearance.

We also observed increased T cell infiltration in the popliteal lymph nodes and ankle joints in PD-1^-/-^ mice following *B*. *burgdorferi* infection, consistent with the role of PD-1/PD-L1 in limiting T cell expansion. One of the major hurdles in assessing these data, however, is the lack of tools to determine T cell antigen specificity to *B*. *burgdorferi*. Tetramers or T cell receptor transgenic T cells specific for *B*. *burgdorferi* antigens would allow us to determine if PD-1 expression was limited to antigen-specific or bystander T cells, and similarly how removal of the PD-1/PD-L1 pathway impacts antigen-specific T cell or bystander T cell expansion. Bystander T cells recognize unrelated antigens but can still expand during infection and disease [52]. During *B*. *burgdorferi* infection in mice, both bystander CD4^+^ and CD8^+^ T cells expand, become activated, and can promote tissue pathology [53]. Future work will aim to address whether PD-1 expression impacts the accumulation and activation of bystander T cells during murine *B*. *burgdorferi* infection.

We chose to study the impact of the PD-1/PD-L1 pathway during *B*. *burgdorferi* infection using the C57BL/6 mouse model of infection because PD-1^-/-^ mice are on the C57BL/6 background. While beneficial from a genetics standpoint, C57BL/6 mice are less susceptible to Lyme arthritis compared to C3H mice [54], and thus phenotypes in the C57BL/6 mice may be masked by using this more resistant model of *B*. *burgdorferi* infection. IL-10^-/-^ mice on the C57BL/6 background have recently become an attractive model for studying Lyme arthritis, as these mice exhibit increased joint inflammation and immune cell infiltration [29]. There is evidence to suggest that there is synergy between IL-10 and the PD-1/PD-L1 pathway during long-term viral infections [55]. IL-10 has been shown to upregulate PD-L1 during HIV infection [56], and given that IL-10 production is induced following *B*. *burgdorferi* stimulation [57], it is reasonable to speculate that immunosuppression driven by IL-10 leads to prolonged *B*. *burgdorferi* loads [29]. Sustained bacterial loads and therefore high amounts of *B*. *burgdorferi* antigen could drive PD-1/PD-L1 T cell mediated exhaustion, as has been described for LCMV infection [58]. In these cases, dual blockade of the PD-1/PD-L1 pathway and IL-10 signaling have enhanced effector T cell functionality, increased infiltration of antigen-specific T cells to the affected tissues, and further reduction in pathogen load [55].

Ultimately, understanding the relative contribution of T cells that express immunoinhibitory molecules to *B*. *burgdorferi* infection and Lyme disease may provide insights into individual variations in responses to the pathogen. It is unknown how PD-1 polymorphisms correlate with patients with long-term Lyme disease symptoms, although there have been studies to suggest that certain polymorphisms in the PD-1 gene in humans are associated with rheumatoid arthritis [59,60]. Interestingly, patients receiving anti-PD-1 or anti PD-L1 immune checkpoint inhibitors for cancer therapy have been shown to spontaneously develop rheumatoid arthritis [61] suggesting these pathways have a role in controlling joint inflammation. However, the contributions of PD-1/PD-L1 in Lyme arthritis in human patients is still unknown. Patients who resolve arthritis symptoms following treatment for Lyme disease, termed antibiotic-responsive Lyme arthritis, have distinct T cell and cytokine profiles from patients who still experience Lyme arthritis symptoms after treatment (post-treatment Lyme arthritis) [62]. Specifically, antibiotic-responsive Lyme arthritis patients experience a higher Treg:effector T cell ratio in synovial fluid compared to patients with post-infectious Lyme arthritis [45]. It is certainly possible that patients with antibiotic-responsive vs. post-treatment Lyme arthritis will experience differential PD-1 expression on Tregs and effector T cells. Future work should aim to uncover whether there is a link between PD-1 upregulation and the resolution of Lyme arthritis following treatment.

Here, we show that the immunoinhibitory pathway comprised of PD-1 and PD-L1 is upregulated during infection and represents a potential mechanism by which *B*. *burgdorferi* evades immune detection. While this pathway is not important for bacterial control, it is important in preventing accumulation of T cells following infection without impacting the B cell response. Further work is needed to understand if other immunoinhibitory pathways are present during infection, how this impacts disease in a more susceptible animal model, and whether T cells from patients suffering from long-term Lyme disease symptoms exhibit differential expression of PD-1 and other immunoinhibitory molecules.

## Materials and methods

### Ethics statement

All mice were housed in the Tufts University Animal facility until use. All experiments were approved by the Tufts Institutional Animal Care and Use Committee (IACUC, Protocol #B2021-84). Euthanasia was performed using CO_2_ asphyxiation followed by cervical dislocation in accordance with guidelines from the American Veterinary Medical Association (AVMA) and was approved by the Tufts IACUC.

### Mice and bacteria

Six- to eight-week old male and female C57BL/6J mice (WT) and B6.Cg-*Pdcd1*^tm1.1Shr^/J mice (PD-1^-/-^) were purchased from The Jackson Laboratory. An infectious isolate of *Borrelia burgdorferi* B31 was cultured in Barbour-Stoenner-Kelley II (BSK-II) medium at 37°C to exponential phase. Bacteria were counted using a Petroff-Hauser counting chamber and used at an MOI = 10 for all *in vitro* experiments. For *in vivo* infections, mice were injected with 100 μl BSK-II (uninfected controls) or 100 μl BSK-II containing 10^5^ *B*. *burgdorferi* subcutaneously in the center of the abdomen. Infection of the mice was validated by culturing the ears of infected mice at the time of sacrifice in BSK-II medium and visualization of spirochetes under a dark-field microscope.

### *In vitro* stimulations

Bone marrow was isolated from WT mouse femurs and tibiae and differentiated into macrophages (BMDMs) by culturing in RPMI containing 10% FBS, 1 mM sodium pyruvate, 10 mM HEPES, and 2 mM L-glutamine (BMDM media) supplemented with 30% L929 cell conditioned medium and an additional 10% FBS for 5 days. Bone marrow derived dendritic cells (BMDCs) were generated by culturing bone marrow for 9 days in RPMI containing 10% FBS, 2 mM L-glutamine, 25 mM HEPES, 50 mM 2-mercaptoethanol, 1X Antibiotic Antimycotic solution (ThermoFisher), and 10 ng/ml murine GM-CSF (Peprotech). Once differentiated, BMDMs or BMDCs were seeded in 6 well plates and stimulated the following day with *B*. *burgdorferi* at an MOI of 10. At 24 hours post-stimulation, cells were removed from the plates using a cell scraper, spun down and washed with MACS buffer (PBS containing 5 mg/ml BSA and 2 mM EDTA).

### Preparation of tissue for flow cytometry

At two, four or eight weeks points post-infection, mice were sacrificed, and the inguinal lymph nodes, popliteal lymph nodes and ankle joints were harvested. Ankle joints were isolated by removing the skin from the lower leg and cutting approximately 3–5 mm above and below the ankle joint. Single-cell suspensions of lymph nodes were prepared by grinding the tissue between frosted microscope slides. Ankle joints were digested in RPMI containing 200 μg/ml Liberase TL (Roche) and 1 U/ml DNase 1 (ThermoFisher) at 37°C for 1 hour, inverting every 15 minutes. Tissues were then dissociated using a transfer pipet, filtered through a 70 μM filter and washed using MACS buffer.

### Flow cytometry

Flow cytometry data were collected on an LSR II (BD Biosciences) and analyzed using FlowJo (Tree Star). All antibodies were purchased from BioLegend except where otherwise noted: CD16/32 (S17011E), CD45 (30-F11), F4/80 (BM8, eBioscience), CD11b (M1/70), CD11c (N418), CD103 (2E7), PD-L1 (10F.9G2), CD3 (17A2), CD4 (GK1.5), CD8α (53–6.7), B220 (RA3-6B2), PD-1 (29F.1A12), T-bet (4B10), Foxp3 (MF-14), CD44 (IM7), CD62L (MEL-14), Gr1 (RB6-8C5), GL7 (GL7), CD95 (Jo2, BD Biosciences), and a LIVE/DEAD Fixable Aqua Dead Cell Stain Kit (Invitrogen). Surface staining was completed in MACS buffer, followed by fixation/permeabilization using a Foxp3 Transcription Factor Staining Buffer Set (Invitrogen) and transcription factor staining in permeabilization buffer. AccuCheck counting beads (Invitrogen) were used to determine absolute cell counts. Following gating by FSC-A and SSC-A, doublets were excluded by gating along the diagonal of FSC-A by FSC-H.

### Cytokine assessment

At specific time points post-infection, ankle joints were excised, flash frozen, and crushed in liquid nitrogen using a mortar and pestle. Protein was extracted by adding 10 μl homogenizing buffer (PBS containing 20 mM Tris HCl (pH 7.5), 0.5% Tween 20, 150 mM NaCl, and 1X Protease Inhibitor Cocktail (ThermoFisher)) per 1 mg tissue weight. Samples were vortexed, spun down for 10 minutes at 10,000 xg, and supernatants isolated and stored at -80°C. Total protein concentration was determined using the Pierce BCA Protein Assay Kit (ThermoFisher). Samples were submitted to Eve Technologies for Mouse Cytokine/Chemokine 31-Plex Discovery Assay and normalized to the total protein concentration after assessment. Cytokine levels only above the limit of detection are reported.

### Droplet digital PCR

Mouse ankle joints, ears and hearts were isolated at specific time points post-infection and DNA was extracted using the DNeasy Blood and Tissue Kit (Qiagen). Droplet digital PCR (ddPCR) was used to quantify the ratio of *Borrelia ospA* to mouse *rpp30* using ddPCR Supermix for Probes (no dUTP) (Bio-Rad) with the following primers and probes (5’ ➔ 3’): *ospA* fwd: ATGTTAGCAGCCTTGACGAG; *ospA* rev: TCGTACTTGCCGTCTTTGTT; *ospA* probe: 6-FAM-AGCGTTTCAGTAGATTTGCCTGGTG-Iowa Black FQ; *rpp30* fwd: CCAGCTCCGTTTGTGATAGT; *rpp30* rev: CAAGGCAGAGATGCCCATAA; *rpp30* probe: HEX-CTGTGCACACATGCATTTGAGAGGT-Iowa Black FQ. Oil-for-Probes droplet emersions were generated using the QX200 droplet generator (Bio-Rad). PCRs were run for 10 minutes at 95°C, 40 cycles of 94°C for 30 seconds and 60°C for 1 minute, followed by 10 minutes at 98°C and subsequent cooling to 4°C. Fluorescent reads were calculated using QX200 droplet reader, analyzed using QX Manager Software (Bio-Rad), and reported as a ratio of copies of *Borrelia ospA* per 1000 copies of mouse *rpp30*.

### Ankle histology

Legs were isolated from WT and PD-1^-/-^ mice at specified time points post-infection. Skin was removed and tissue was placed in Bouin’s solution for one week to decalcify the bone. Tissues were then paraffin embedded, sectioned and stained with hematoxylin and eosin (H&E) at the Rodent Histopathology Core at Harvard Medical School or the Department of Comparative Pathobiology at the Tufts Cummings School of Veterinary Medicine. Slides were blindly scored by the Tufts Veterinary Pathologist using the scoring system outlined in S8 Fig.

### Anti-*Borrelia* ELISA

Sera were collected from WT and PD-1^-/-^ mice at time of sacrifice and stored at -80°C until use. To detect anti-*Borrelia* antibodies, 96-well plates (EIA/RIA, Corning) were coated with 5 μg/ml sonicated *B*. *burgdorferi* in PBS overnight at 4°C. Plates were then blocked for two hours at room temperature with 1% bovine serum albumin (BSA) in PBS. Sera were diluted 1:5000 in PBS and incubated on plates for 3 hours at room temperature. Plates were then incubated with anti-mouse HRP-linked IgG secondary antibody (ThermoFisher) at a dilution of 1:2000 in PBS containing 1% BSA for 1 hour at room temperature and developed with Sure Blue TMB peroxidase substrate followed by addition of equal volume of 1N HCl. Absorbance was measured at 450 nm.

### Statistical analysis

Data were analyzed using Prism software. Differences were considered statistically significant if the *P* value was less than 0.05. All data are graphed as the means ± standard errors of the means (SEM). For all figures, * *P* < 0.05, ** *P* < 0.01, *** *P* < 0.001, and **** *P* < 0.0001.

## Supporting information

S1 Fig Flow cytometry gating of bone marrow derived macrophages and dendritic cells. (A) BMDMs were determined as PD-L1^+^ by gating on singlets ➔ live ➔ CD45^+^ F4/80^+^ ➔ PD-L1^+^ cells. (B) BMDCs were determined as PD-L1^+^ by gating on singlets ➔ live ➔ CD45^+^ CD11c^+^ ➔ CD103^+/-^ ➔ CD11b^-/+^ ➔ PD-L1^+^.
(PDF)

S2 Fig Flow cytometry gating of antigen presenting cell subsets and PD-L1 expression in the inguinal lymph node. (A) PD-L1 on macrophages was assessed by gating on live ➔ CD45^+^ ➔ CD11c^-^ ➔ F4/80^+^ ➔ PD-L1^+^ cells, while dendritic cells were assessed by live ➔ CD45^+^ ➔ CD11c^+^ ➔ CD103^+/-^ CD11b^-/+^ ➔ PD-L1^+^ cells. (B) Inguinal lymph nodes were harvested from uninfected mice and at week two and four post-infection and F4/80^+^ macrophages, CD11b^+^ CD103^-^ dendritic cells and CD11b^-^ CD103^+^ dendritic cells were assessed for PD-L1 expression by flow cytometry. Data are pooled from two independent experiments with at least six mice per group and were analyzed using two-way ANOVA with Dunnett’s multiple comparisons test.
(PDF)

S3 Fig CD8^+^ T cells and B cells do not upregulate PD-1 following *B*. *burgdorferi* infection. (A) Representative flow cytometry gating of PD-1^+^ B cells and CD8^+^ T cells through gating on singlets ➔ live ➔ CD45^+^ ➔ B220^+^ or CD3^+^ ➔ PD-1^+^ (B cells) or CD8^+^ ➔ PD-1^+^ cells. At two, four, and eight weeks post-infection, (B) popliteal lymph nodes and (C) ankle joints were assessed for PD-1 expression on CD8^+^ T cells and B cells by flow cytometry. (D) Inguinal lymph nodes were assessed for PD-1 expression on CD4^+^ T cells, CD8^+^ T cells, and B cells by flow cytometry. Data are representative of four independent experiments, shown with five mice per group and were analyzed using ordinary one-way ANOVA with Dunnett’s multiple comparisons test.
(PDF)

S4 Fig The PD-1/PD-L1 pathway does not impact bacterial load in the ear and heart during *B*. *burgdorferi* infection. WT and PD-1^-/-^ mice were inoculated with *B*. *burgdorferi* or media alone and sacrificed at two, four, and eight weeks post-infection. DNA was isolated from (A) the ear and (B) the heart and bacterial load was assessed by ddPCR. Data are representative of three experiments pooled with at least five mice per group and were analyzed using two-way ANOVA with Sidak’s multiple comparisons test.
(PDF)

S5 Fig Inflammatory cytokine and chemokine analysis of ankle joints reveals increased CCL4 and CXCL1 levels in the absence of PD-1. WT and PD-1^-/-^ mice were inoculated with *B*. *burgdorferi* or media alone and sacrificed two and four weeks post-infection. Protein was extracted from ankle joints and assessed for the presence of 31 different inflammatory cytokines. (A) Cytokine levels in ankle joints above the level of detection. (B) CCL4 expression and (C) CXCL1 expression in the ankle joints over time. Data are representative of at least four mice per group (three for each uninfected group) and (B) and (C) were analyzed using two-way ANOVA with Sidak’s multiple comparisons test.
(PDF)

S6 Fig PD-1^-/-^ mice exhibit increased infiltration of macrophages and neutrophils to the popliteal lymph nodes following infection. WT and PD-1^-/-^ mice were inoculated with *B*. *burgdorferi* or media alone and sacrificed two, four, and eight weeks post-infection. Absolute numbers of macrophages were assessed in the (A) popliteal lymph nodes, (C) inguinal lymph nodes and (E) ankle joints by gating on live ➔ CD45^+^ CD3^-^ ➔ CD11b^+^ F4/80^+^ cells. Absolute numbers of neutrophils were assessed in the (B) popliteal lymph nodes, (D) inguinal lymph nodes and (F) ankle joints by gating on live ➔ CD45^+^ CD3^-^ ➔ CD11b^+^ Gr1^hi^ cells. Data are pooled from four independent experiments with at least five mice per group and were analyzed using two-way ANOVA with Sidak’s multiple comparisons test.
(PDF)

S7 Fig PD-1 does not influence the T cell and B cell responses in the inguinal lymph nodes. WT and PD-1^-/-^ mice were inoculated with *B*. *burgdorferi* or media alone and inguinal lymph nodes were isolated at two, four, or eight weeks post-infection. (A) CD4^+^ T cells, (B) CD8^+^ T cells, (C) B cells, and (D) germinal center B cells were determined by flow cytometry. Data are pooled from at least two independent experiments with five mice per group and were analyzed using two-way ANOVA with Sidak’s multiple comparisons test.
(PDF)

S8 Fig Histopathology scoring system used to assess ankle inflammation. (PDF)
