# Supplementary figures and images for: The PD-1/PD-L1 pathway is induced during Borrelia burgdorferi infection and inhibits T cell joint infiltration without compromising bacterial clearance
Source: PLoS Pathog. 2022 Oct 20;18(10):e1010903. doi: 10.1371/journal.ppat.1010903 (PMC9624412; doi:10.1371/journal.ppat.1010903)

**A**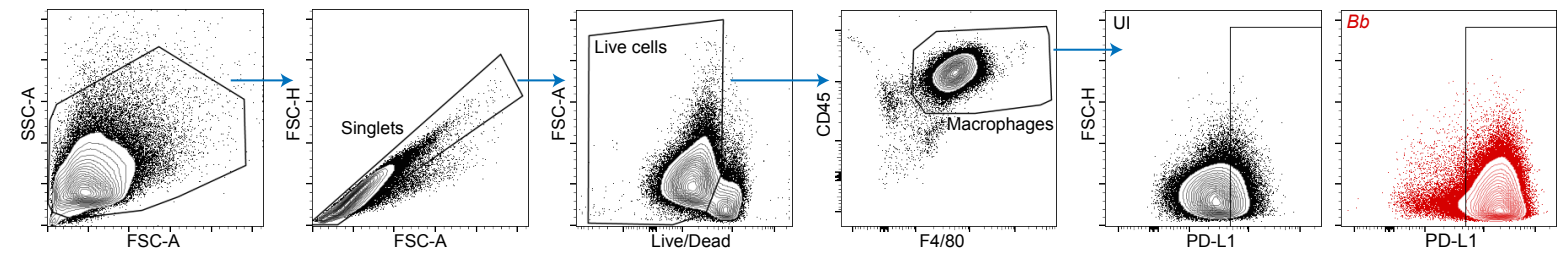**B**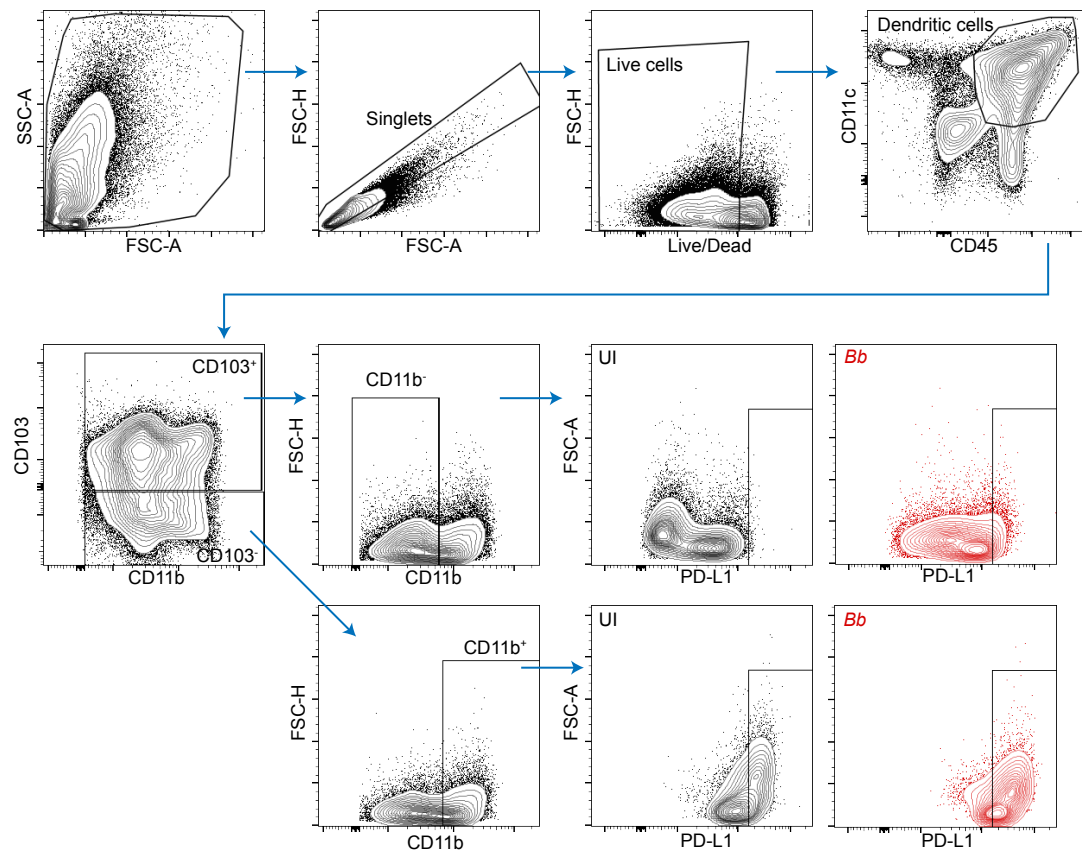

Supplement: S1 Fig — (A) BMDMs were determined as PD-L1+ by gating on singlets ➔ live ➔ CD45+ F4/80+ ➔ PD-L1+ cells. (B) BMDCs were determined as PD-L1+ by gating on singlets ➔ live ➔ CD45+ CD11c+ ➔ CD103+/- ➔ CD11b-/+ ➔ PD-L1+. (PDF) [file ppat.1010903.s001.pdf]

**A**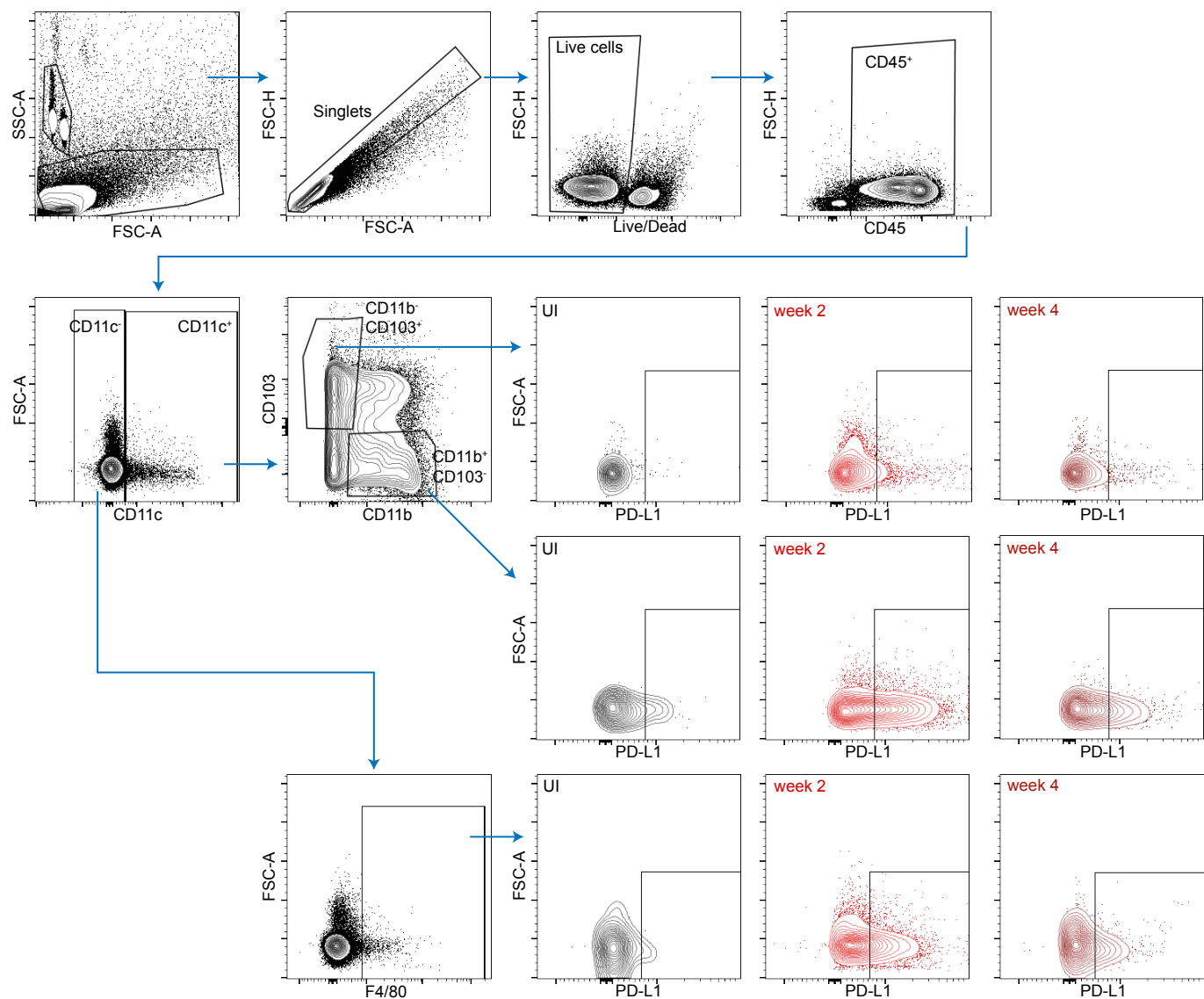**B**

### Inguinal lymph node

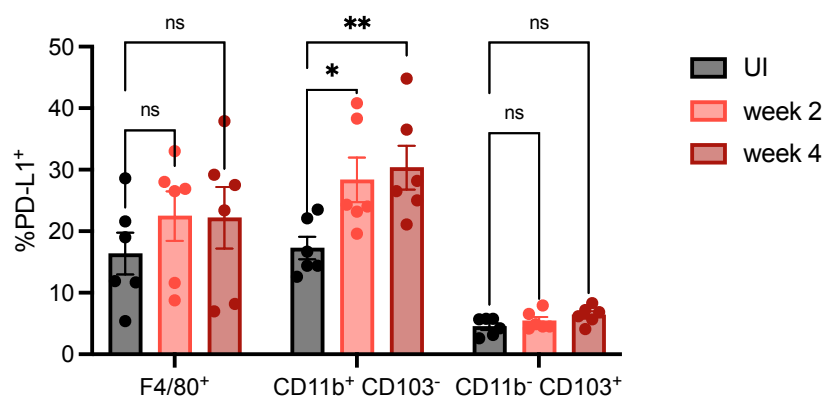

Supplement: S2 Fig — (A) PD-L1 on macrophages was assessed by gating on live ➔ CD45+ ➔ CD11c- ➔ F4/80+ ➔ PD-L1+ cells, while dendritic cells were assessed by live ➔ CD45+ ➔ CD11c+ ➔ CD103+/- CD11b-/+ ➔ PD-L1+ cells. (B) Inguinal lymph nodes were harvested from uninfected mice and at week two and four post-infection and F4/80+ macrophages, CD11b+ CD103- dendritic cells and CD11b- CD103+ dendritic cells were assessed for PD-L1 expression by flow cytometry. Data are pooled from two independent experiments with at least six mice per group and were analyzed using two-way ANOVA with Dunnett’s multiple comparisons test. (PDF) [file ppat.1010903.s002.pdf]

**A**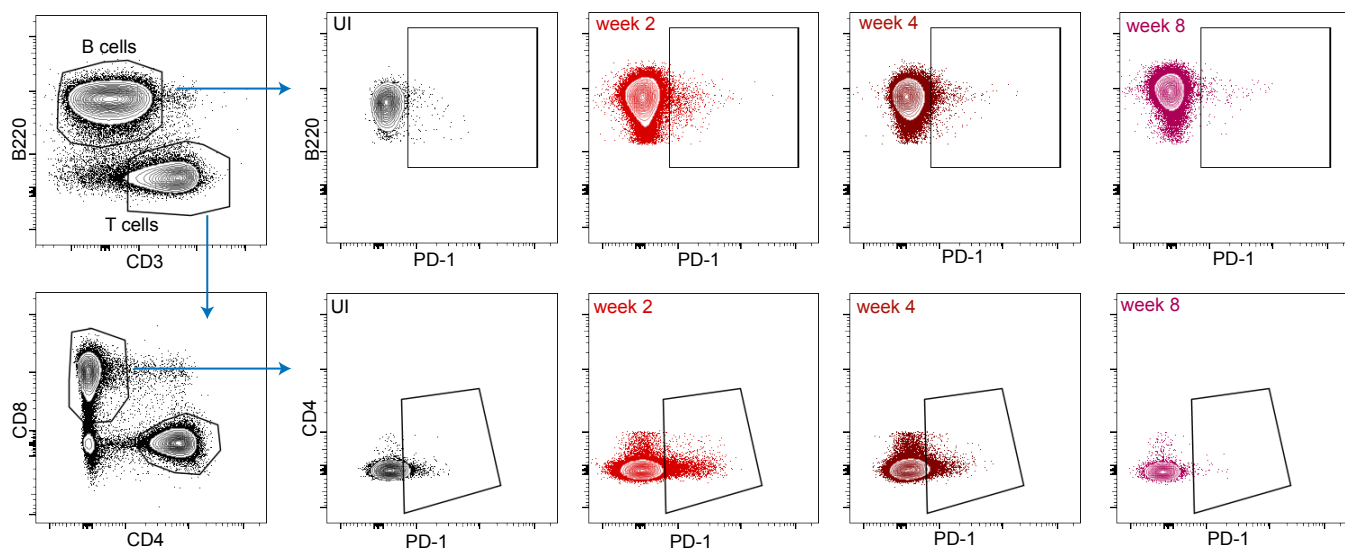**B**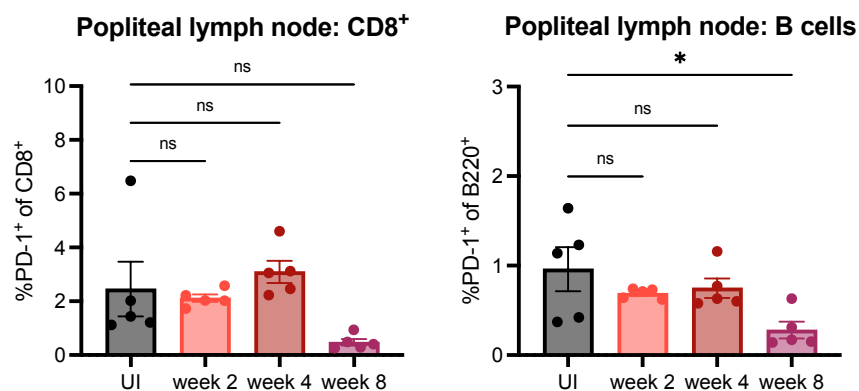**C**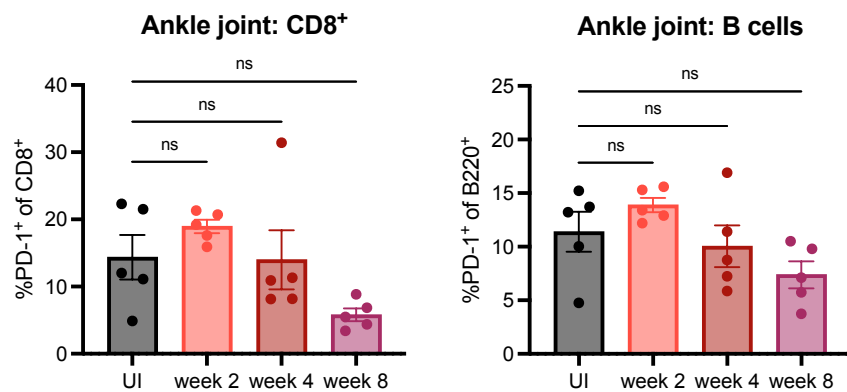**D**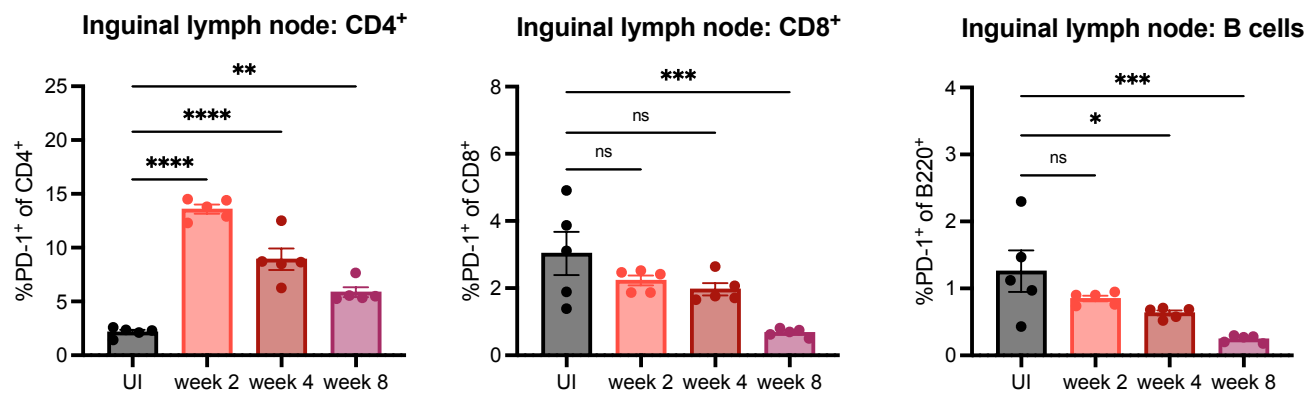

Supplement: S3 Fig — (A) Representative flow cytometry gating of PD-1+ B cells and CD8+ T cells through gating on singlets ➔ live ➔ CD45+ ➔ B220+ or CD3+ ➔ PD-1+ (B cells) or CD8+ ➔ PD-1+ cells. At two, four, and eight weeks post-infection, (B) popliteal lymph nodes and (C) ankle joints were assessed for PD-1 expression on CD8+ T cells and B cells by flow cytometry. (D) Inguinal lymph nodes were assessed for PD-1 expression on CD4+ T cells, CD8+ T cells, and B cells by flow cytometry. Data are representative of four independent experiments, shown with five mice per group and were analyzed using ordinary one-way ANOVA with Dunnett’s multiple comparisons test. (PDF) [file ppat.1010903.s003.pdf]

**A****Ear: bacterial load**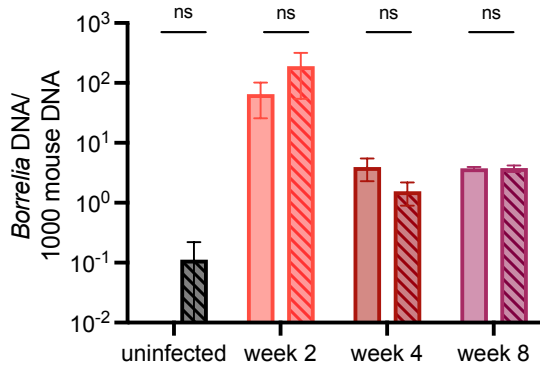**B****Heart: bacterial load**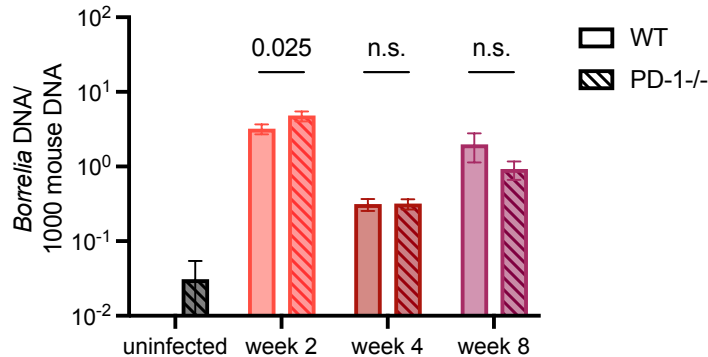

Supplement: S4 Fig — WT and PD-1-/- mice were inoculated with B. burgdorferi or media alone and sacrificed at two, four, and eight weeks post-infection. DNA was isolated from (A) the ear and (B) the heart and bacterial load was assessed by ddPCR. Data are representative of three experiments pooled with at least five mice per group and were analyzed using two-way ANOVA with Sidak’s multiple comparisons test. (PDF) [file ppat.1010903.s004.pdf]

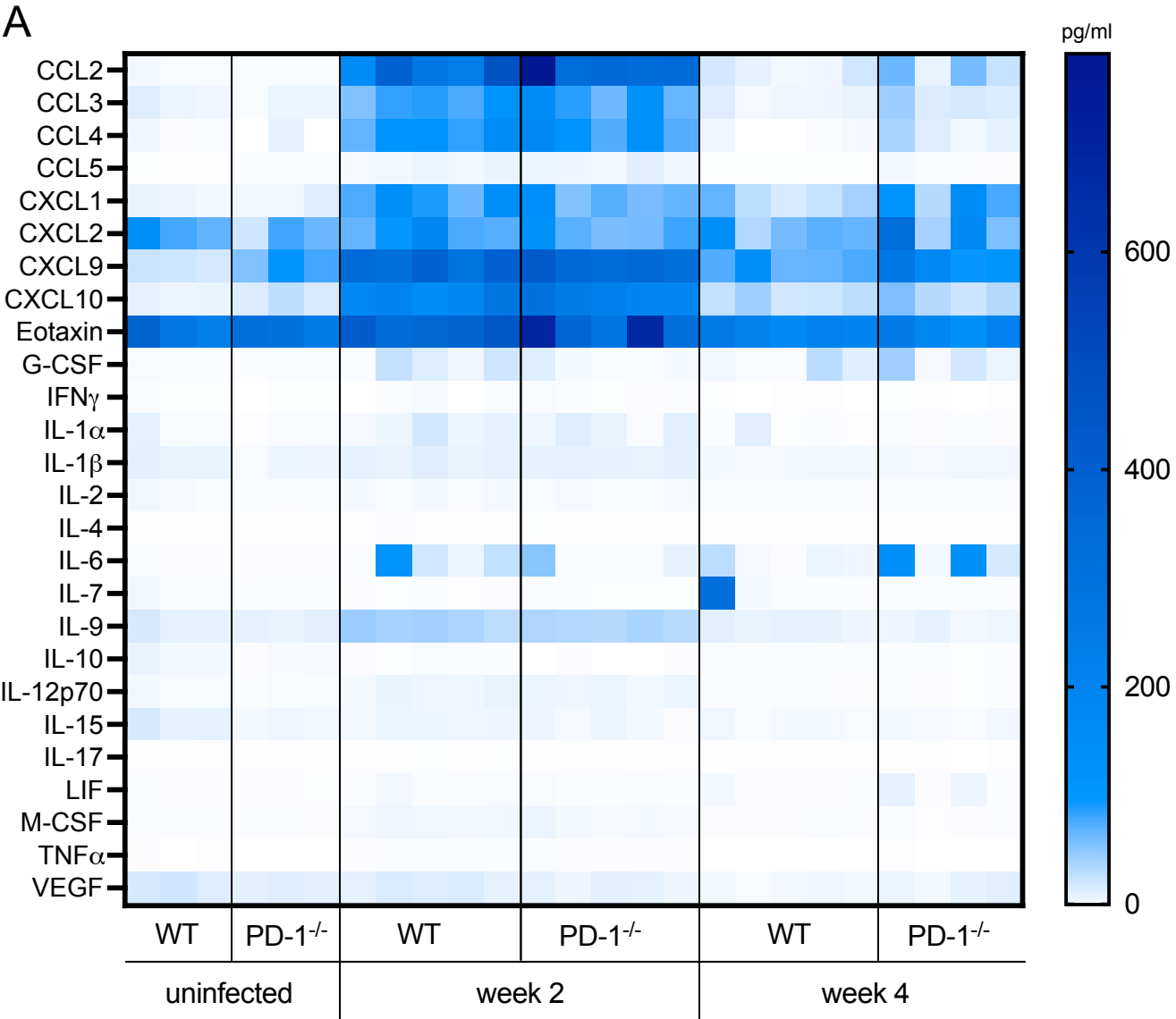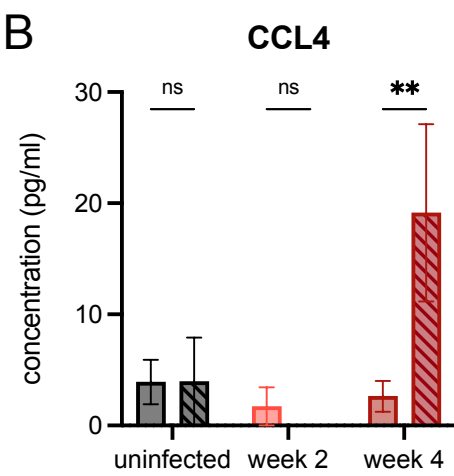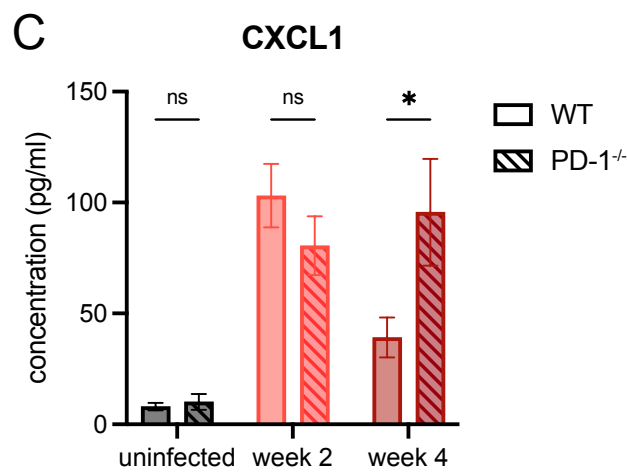

Supplement: S5 Fig — WT and PD-1-/- mice were inoculated with B. burgdorferi or media alone and sacrificed two and four weeks post-infection. Protein was extracted from ankle joints and assessed for the presence of 31 different inflammatory cytokines. (A) Cytokine levels in ankle joints above the level of detection. (B) CCL4 expression and (C) CXCL1 expression in the ankle joints over time. Data are representative of at least four mice per group (three for each uninfected group) and (B) and (C) were analyzed using two-way ANOVA with Sidak’s multiple comparisons test. (PDF) [file ppat.1010903.s005.pdf]

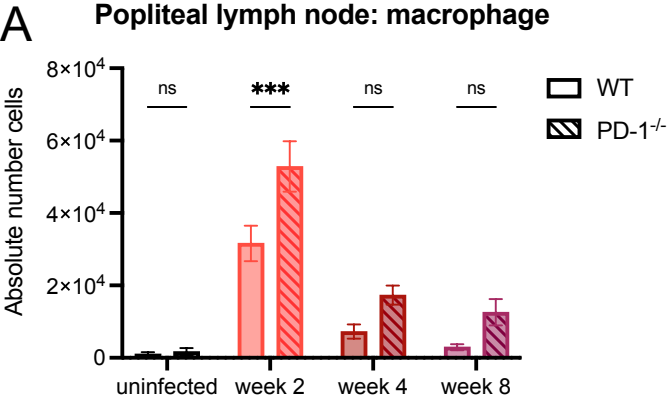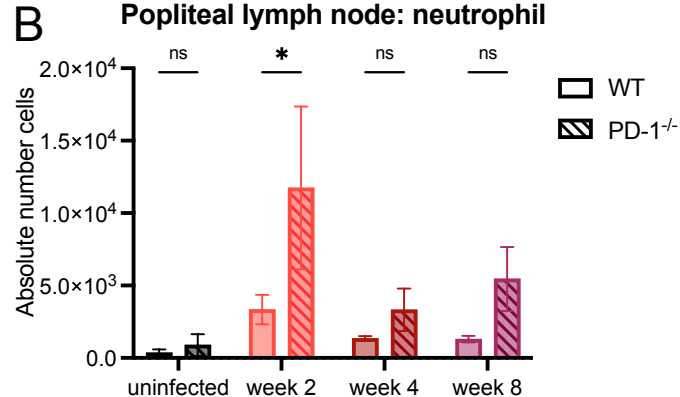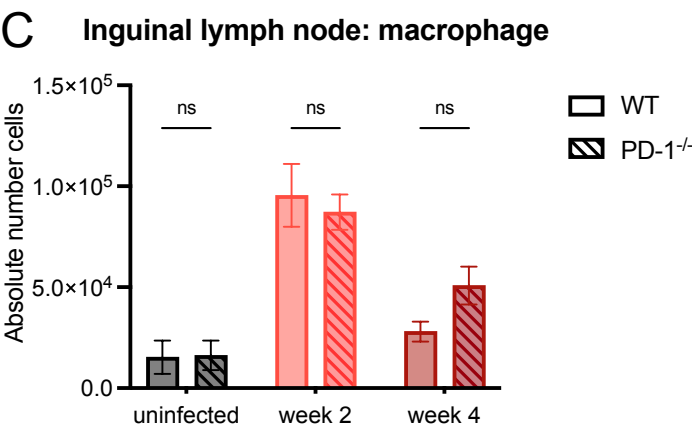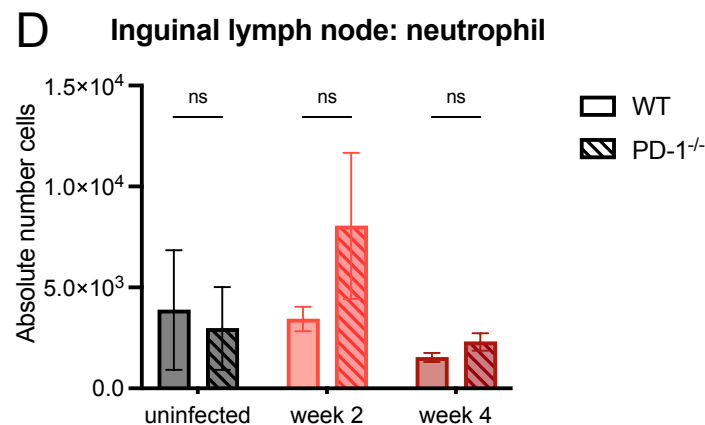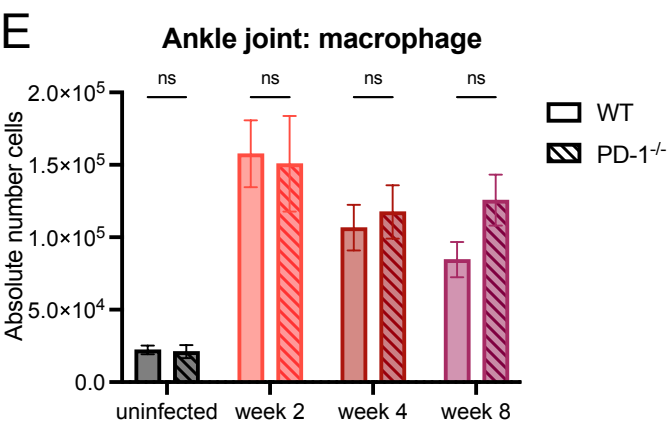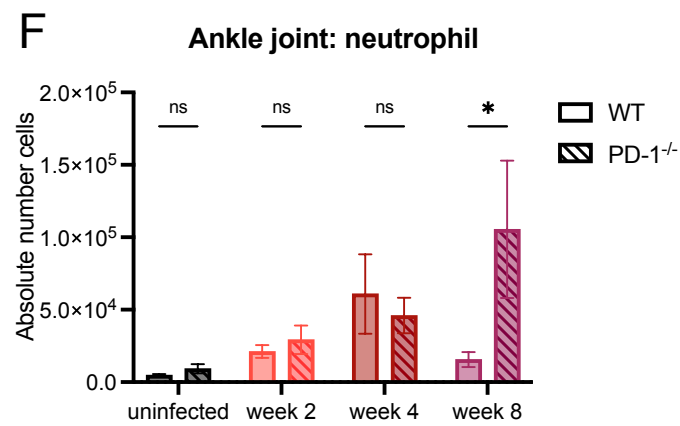

Supplement: S6 Fig — WT and PD-1-/- mice were inoculated with B. burgdorferi or media alone and sacrificed two, four, and eight weeks post-infection. Absolute numbers of macrophages were assessed in the (A) popliteal lymph nodes, (C) inguinal lymph nodes and (E) ankle joints by gating on live ➔ CD45+ CD3- ➔ CD11b+ F4/80+ cells. Absolute numbers of neutrophils were assessed in the (B) popliteal lymph nodes, (D) inguinal lymph nodes and (F) ankle joints by gating on live ➔ CD45+ CD3- ➔ CD11b+ Gr1hi cells. Data are pooled from four independent experiments with at least five mice per group and were analyzed using two-way ANOVA with Sidak’s multiple comparisons test. (PDF) [file ppat.1010903.s006.pdf]
