# Supplementary material for: The PD-1/PD-L1 pathway is induced during Borrelia burgdorferi infection and inhibits T cell joint infiltration without compromising bacterial clearance
Source: PLoS Pathog. 2022 Oct 20;18(10):e1010903. doi: 10.1371/journal.ppat.1010903 (PMC9624412; doi:10.1371/journal.ppat.1010903)
Supplement: S7 Fig — WT and PD-1-/- mice were inoculated with B. burgdorferi or media alone and inguinal lymph nodes were isolated at two, four, or eight weeks post-infection. (A) CD4+ T cells, (B) CD8+ T cells, (C) B cells, and (D) germinal center B cells were determined by flow cytometry. Data are pooled from at least two independent experiments with five mice per group and were analyzed using two-way ANOVA with Sidak’s multiple comparisons test. (PDF) [file ppat.1010903.s007.pdf]

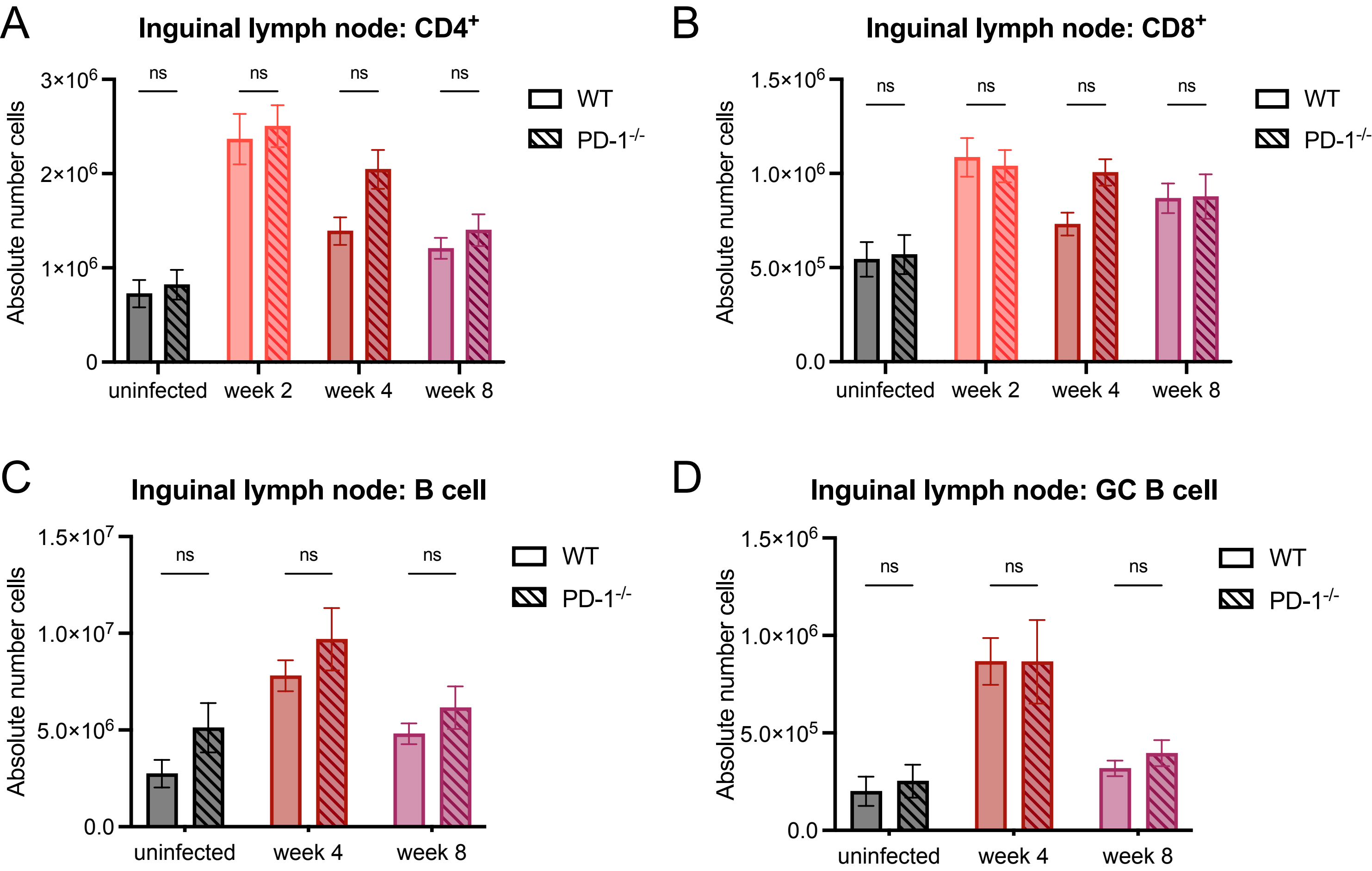
A
Inguinal lymph node: CD4+
Absolute number cells
3×10^6
2×10^6
1×10^6
0
ns
ns
ns
ns
uninfected
week 2
week 4
week 8
WT
PD-1-/-
B
Inguinal lymph node: CD8+
Absolute number cells
1.5×10^6
1.0×10^6
5.0×10^5
0.0
ns
ns
ns
ns
uninfected
week 2
week 4
week 8
WT
PD-1-/-
C
Inguinal lymph node: B cell
Absolute number cells
1.5×10^7
1.0×10^7
5.0×10^6
0.0
ns
ns
ns
uninfected
week 4
week 8
WT
PD-1-/-
D
Inguinal lymph node: GC B cell
Absolute number cells
1.5×10^6
1.0×10^6
5.0×10^5
0.0
ns
ns
ns
uninfected
week 4
week 8
WT
PD-1-/-
