# Supplementary material for: The PD-1/PD-L1 pathway is induced during Borrelia burgdorferi infection and inhibits T cell joint infiltration without compromising bacterial clearance
Source: PLoS Pathog. 2022 Oct 20;18(10):e1010903. doi: 10.1371/journal.ppat.1010903 (PMC9624412; doi:10.1371/journal.ppat.1010903)
Supplement: S8 Fig — (PDF) [file ppat.1010903.s008.pdf]

Synovitis and Tenosynovitis/Tendonitis
0 = No inflammation
1 = <10% of the joint space or tendon sheath and synovium infiltrated by mononuclear leukocytes and/or neutrophils
2 = 10 - 33%
3 = 33 - 66%
4 = >66%

Periarticular Fibrosis
0 = No fibrosis
1 = <10% of periarticular region replaced by fibrosis (streams of dense, irregular connective tissue and fibroblasts)
2 = 10 - 33%
3 = 33 - 66%
4 = >66%

Synovial Proliferaton
0 = Normal synovium
1 = Synovial hyperplasia (synoviocytes that are plump, cuboidal and piled in multiple, disorganized layers on papillary projections)

Articular Cartilage Degeneration or Erosion
0 = Normal articular cartilage
1 = Cartilage degeneration and necrosis (loss of or hypereosinophilic cartilage matrix, chondrocyte necrosis)

Periosteal Remodeling
0 = No remodeling
1 = Bone remodeling (scalloped edges lined by osteoclasts with deposition of woven bone)

Effusion/Edema
0 = No effusion or periarticular edema
1 = Effusion (lightly amorphous, wispy to amporphous material within or around joint space or tendon sheath)

Approach: Two ankle sections are present on each slide (presumed bisected leg). For each criterion, the total tissue field present on the slide is examined, and the percent affected or presence of the lesion is evaluated. Total possible score per joint: 16.
